# Inculcation of Green Behavior in Employees: A Multilevel Moderated Mediation Approach

**DOI:** 10.3390/ijerph18010331

**Published:** 2021-01-05

**Authors:** Maria Saleem, Faisal Qadeer, Faisal Mahmood, Heesup Han, Gabriele Giorgi, Antonio Ariza-Montes

**Affiliations:** 1 Lahore Business School, The University of Lahore, Lahore 54000, Pakistan; mariyasaleem1989@gmail.com (M.S.); mfaisalqr@gmail.com (F.Q.); faisalch62@gmail.com (F.M.); 2 Department of Hospitality and Tourism Management, Sejong University, Seoul 05006, Korea; heesup.han@gmail.com; 3 Department of Human Sciences, Università Europea di Roma, 00163 Roma, Italy; gabriele.giorgi@unier.it; 4 Social Matters Research Group, Universidad Loyola Andalucía, 14004 Córdoba, Spain; 5 Facultad de Administración y Negocios, Universidad Autónoma de Chile, Santiago 425, Chile

**Keywords:** ethical leadership, employee green behavior, social learning theory, green psychological climate, leaders’ pro-environmental attitudes, employees’ harmonious environmental passion, employees’ environmental commitment, multi-level

## Abstract

In this era of globalization, preventing organizations from undermining and degrading the environment has become a great challenge, especially when considering that organizations are among the major contributors to environmental deterioration. As a result, scholars have recently begun to focus on understanding the key determinants of employee green behavior (EGB), a nascent field within the area of sustainable development and organizational behavior. This study extends the emerging discussion over EGB by investigating how green behavior can be inculcated into employees’ mindsets and under what conditions this can best be accomplished. The present research examines the relationship between ethical leadership and EGB by the mediating mechanisms of green psychological climate, employees’ harmonious environmental passion, and employees’ environmental commitment, through the underpinnings of social learning theory. Further, the study examines the contingency effects of leaders’ pro-environmental attitudes to determine how leaders with ethical attributes and pro-environmental attitudes can create a green psychological climate that ultimately leads to EGB through employees’ harmonious environmental passion and employees’ environmental commitment. The approach to implementing theory development is deductive as the research employed a quantitative research design and survey administration with a time-lagged approach. Multi-level data were collected from 400 respondents working in public and private sector hospitals and universities in Pakistan. The analysis was conducted in MPlus. The results show positive and statistically significant effects of ethical leadership on EGB through the serial mediations of a green psychological climate and employees’ harmonious environmental passion, and a green psychological climate and employees’ environmental commitment. Moreover, the leaders’ pro-environmental attitude contingency strengthens the indirect impact of ethical leadership on EGB. This research provides several managerial implications through which organizations can strategically concentrate on EGB, including saving energy by turning off unused lights, reducing waste, and recycling.

## 1. Introduction

The global population is increasing every day [1] and uses natural resources at a rate that cannot be sustained indefinitely. Coinciding with this increased use of natural resources, researchers observe that the environment is experiencing numerous changes; for instance, there has been an increase in the average global ocean and air temperatures. The reduction of glaciers and increased mean sea level is another sign of this changing environment [2]. In today’s globalized world, the association between and collaboration among corporations and environmental safety organizations have become increasingly important and are being considered by academicians, practitioners, and society as a whole [3]. It has been illustrated that climate changes result from various human activities, such as increasing greenhouse gases [4]. Hence, to achieve a more sustainable means of life, there is a need for intense changes in human activity. These changes are to prevent devastating environmental consequences. During the last part of the 20th century, there was an emerging debate regarding sustainability issues. Sustainability is an essential fundamental component of the world’s social and economic development [5]. Sustainable development is the “development that meets the needs of the present without compromising the ability of future generations to meet their own needs” [6] (p. 43). Bonan [7] proclaimed that conventional economic thinking has substantially contributed to climate change. There must be exemplary changes in science, technology, and human behavior to move towards sustainable growth and development [8].

This article aims to discuss how environmental sustainability is relevant to organizations. Employees’ perception concerning their organization’s context is a major contributing factor to employee behavior and performance. In this sense, how organizations are managing the challenge of environmental sustainability is critical. Academicians have argued that organizations possess the power to make the necessary changes to improve the environment [9]. In this same vein, all well-established resources and major stakeholder theories hold that sustainability makes good business sense [10]. Consequently, an organization’s environmental performance is regarded as an essential aspect to determine that organizations’ overall performance in the 21st century [11].

Many organizations portray their environmental commitment via different certifications [12]. Although technological advancements are considered positive, the empirical confirmation advocates that they are a weak kind of sustainability [8]. These technological advancements may not guarantee the achievement of environmental goals and outcomes [13]. For instance, it has been argued in the literature that these types of certifications are based only on assessments of an environmental management system, indicating whether or not a system is present in the organization. This coverage may not accurately portray the organization’s environmental performance or its impact [12]. Russell and McIntosh [14] suggested that it is vital to train and encourage employees to promote sustainability as a signal of a pro-active approach towards sustainability and technology. Accordingly, implementing an organization’s environmental management system depends on human resources and environmentally friendly practices [15]. Hence, to shape employees’ behaviors towards more environmentally sustainable practices is essential. Because employees play critical roles in protecting the environment, organizations should take a considerable interest in engaging employees in sustainable movements [16]. Hence, it is crucial to research the factors required to encourage employees to adopt green behaviors.

Given the valuable consequences of EGB, various attempts have been made to discover its antecedents. For instance, some predetermining factors are knowledge and awareness [17], eco-friendly specific servant leadership [18], and human resource management practices [19]. Further, the present literature has established various mechanisms to investigate employees’ engagement in green behaviors [20]. However, the research on the determinants of EGB is yet in its emergence [18,19,21], and this infancy is even less advanced in developing countries, for instance, Pakistan. Therefore, comprehension of the mechanisms by which organizations can inculcate EGB is incomplete and limited [21]. Existing research remains deficient for contingencies and theoretical considerations of how organizations’ environmental practices and policies are related to EGB [21]. Moreover, the existing literature’s deficiency is also noted concerning the immediate leader’s role in encouraging employees to engage in green behaviors [22]. Accordingly, there are increasing calls to investigate how ethical leadership can impact followers’ perceptions to influence their workplace behaviors [23]. Furthermore, few studies have investigated how green behaviors can be promoted in the workplace [22,24]. Notably, most of the research on EGB is theoretical, and few studies have empirically examined EGB. Therefore, the present research endeavors to fill these gaps by empirically investigating, from a multi-level perspective, the determinants of EGB that differ from the factors previously identified in the literature.

This study uses an ambitious and unusual design to investigate how and under what boundary conditions ethical leadership can inculcate EGB in organizations. Ethical leadership seems to be appropriate here in contrast to other moral/ethical based leadership styles, including servant, responsive, and authentic leadership. Ethical leadership’s critical aspects are fairness, role clarification, power-sharing, people-orientation, ethical guidance, integrity, and concern for sustainability [25]. In contrast, authentic leadership includes self-awareness, moral perspectives, relational transparency, balanced processing, and authentic behavior [26]. In comparison, servant leadership’s key characteristics are empathy, listening, healing, persuasion, awareness, concentration on others’ growth, and the community [27]. Responsive leadership is “the process that leads to building and sustaining positive relationships with both internal and external stakeholders to the organization” [28]. Mayer et al. [29] noted that ethical leadership is different from the other moral/ethical based leadership styles. It concentrates on the ethical aspect of leadership instead of incorporating ethics as an auxiliary aspect. Ethical leadership comprises traits such as “moral person”, and behavior, such as “moral manager”. It is also explicated that ethical leadership is indicated by the leaders’ traits, for instance, fairness and integrity [30]. An ethical leader seeks to accomplish the right thing and performs leadership roles in an ethical manner [31]. Furthermore, ethical leadership underpins Bandura’s [32] social learning theory and asserts that an ethical leader prompts followers to become involved in ethical behaviors by behavioral modeling and through communication, reward, and punishment [26]. In a similar vein, Brown and Treviño [31] clarified that ethics are an essential element for effective leadership, and leaders are liable to promote ethical climates and behaviors.

This study relies on multi-level and multisource data collected by employing a time-lag design to extend the field. Furthermore, this study alters the already ongoing EGB debate and considerably extends the present literature in several ways. For instance, this research investigates an aspiring framework that strives to offer a comprehensive understanding of EGB inculcation. The underlying framework is said to be ambitious because it considers the effect of group-level ethical leadership on the green psychological climate at the employee level, which further translates into employees’ harmonious environmental passion and employees’ environmental commitment, which in turn supports EGB. Additionally, the boundary condition of group-level leaders’ pro-environmental attitude enhances the scope of this research.

Concerning the empirical context, the present research provides numerous insights for organizations and managers. Organizational leaders and employees can play an essential role in achieving organizations’ sustainability and environmental protection goals. In this regard, the inculcation of green employee behavior seems to be worthwhile. Organizational leaders’ role is to promote eco-friendly behavior through role modeling and personal behavior concerning the natural environment. In public health and academic institutions, sustainable development is urgently needed due to excessive use of paper, recycling and reusable materials, electricity, and energy usage. Thus, the concentration on leaders’ and employees’ eco-friendly behaviors in these organizations becomes indispensable so that environmental decay and depletion of natural resources can be minimized.

## 2. Hypotheses Development

### 2.1. Social Learning Theory

This is a theory of social behavior and of the learning process that intends that new behaviors can be learned by imitating and observing others. It explains that learning is a cognitive procedure that happens in a social setting and can occur solely by observation or by direct coaching, even in the lack of direct reinforcement. Apart from the observation of behavior, learning also happens by the observation of punishments and rewards, a procedure recognized as vicarious reinforcement [30,31,32]. This theory argues that individuals look externally to themselves for ethical guidelines and ultimately learn from ethical and credible role models [31]. Additionally, social learning theory includes the effect of a solo role model on a solo person and elaborates on the collective context. The literature argues that an ethical climate represents the collective or group and team level aspects of social learning theory. In this context, the role of vicarious learning is highlighted by the researchers, in which individuals learn to behave appropriately with the help of the prevailing norms of social contexts [33].

### 2.2. Mediation of a Green Psychological Climate on the Relationship of Ethical Leadership and Employees’ Harmonious Environmental Passion

Ethical leadership is “the demonstration of normatively appropriate conduct through personal actions and interpersonal relationships and his or her promotion of such conduct to followers through two-way communication, reinforcement, and decision making” [30]. Ethical leadership is strongly linked with employees’ ethical behaviors, and it positively affects the ethical conducts and attitudes of employees in a way that ultimately improves organizational performance [21,34,35]. The green psychological climate is “the employees’ perceptions and interpretations of their organization’s policies, procedures, and practices regarding environmental sustainability” [21,36]. Before acting upon the policies promoted in their work setting, employees must realize and clarify these policies [36]. At all organizational levels, leaders perform significant roles to shape organizational climate [37]. Employees will generally follow the instructions specified by management and go in the similar way that management eventually goes. Accordingly, leaders and top management can create a suitable work climate if they promote the green behaviors that they want their employees to display [38].

Thus, an ethical leader plays a significant role in shaping employees’ behaviors by acting as a role model. Moreover, leadership can impart the importance and significance of sustainability issues [39]. Leaders can evoke emotional responses in followers, and employees’ harmonious environmental passion is a positive emotion that prompts eco-friendly behaviors, referred as an affirmative emotion, so that employees’ harmonious environmental passion results in an individual’s desire to be involved in pro-environmental activities [22]. Employees are usually like to perform and be more excited about the kinds of behaviors that are the most vital for the organization and that are perceived as having social importance. Thus, vibrant indications must be given to the employees, and a vision that expresses the significance of the ecological sustainability aims of the workplace and of its leaders should be voiced by the organization [22]. Employees’ understandings of these sustainable environmental goals, policies, and procedures create a green psychological climate in organizations. Furthermore, when they observe their leaders passionately performing green behaviors, and the efforts of their leaders in making the climate of the organization greener, employees strive to harmonize with the actions and attitudes of their leaders. This reaction is an emotional contagion that occurs between leaders and followers and is considered a key component of the leadership process [40]. Hence, it is this emotional contagion that ignites employees’ harmonious environmental passion [22].

Depending on the creeds of social learning theory, employees’ thoughts, beliefs, attitudes, and behaviors can be potentially influenced by the climate. Ethical leaders are considered the moral managers who affect the groups’ moral climate and represent an ethical role model to follow [41]. Thus, it is entirely reasonable to argue that ethical leaders’ green psychological climate ultimately results in employees’ harmonious environmental passion. Role modeling by leaders is likely to influence the climate by displaying behaviors that align with their words [42]. When employees’ observe their leaders’ efforts in making the climate of the organization greener, employees strive to harmonize with the actions and attitudes of their leaders which translates into employees’ harmonious environmental passion. Thus, the following hypothesis is proposed:

**Hypothesis** **1.** *The relationship between ethical leadership and employees’ harmonious environmental passion is mediated by green psychological climate*.

### 2.3. Mediation of a Green Psychological Climate on Ethical Leadership and Employees’ Environmental Commitment Relationship

The research regarding ethical leadership has concentrated on the positive and negative effects of ethical leadership on employee behaviors. Hoch et al. [26] in their meta-analysis noted that ethical leadership increases desired employee outcomes like job satisfaction, organizational commitment, employee engagement, and organizational citizenship behavior, while simultaneously playing a vital role in reducing employee deviance and turnover intention. Ethical leadership is also strongly linked with employees’ ethical behaviors and positively affects employees’ attitudes and ethical conduct, thereby improving organizational performance [25,34]. As ethical leadership improves the psychological climate within an organization [43], it has been argued that, when the leader is highly ethical, the ethical climate in the organization will increase [44]. In this way, the climate leads to collective adoption by employees of the organization’s ethical policies, practices, and procedures [44]. The employees’ environmental commitment is “an internal, obligation-based motivation and as having emotional attachment, identification, and involvement with environmental behaviors” [45,46]. Moreover, Raineri and Paillé [47] defined it as a feeling of attachment and a feeling of being responsible for environmental issues in the workplace. Commitment is said to be a mindset or psychological state that guides behaviors towards a specific target.

Social learning theory (SLT) posits that employees can learn suitable behaviors via role modeling and the use of reinforcement [22]. It seems that ethical leaders are eagerly observable. Moreover, they model appropriate and suitable behaviors. Ethical standards are effectively being communicated by them, along with compliance with ethical norms and behavior, and with certain criteria of rewards and punishments for their employees. Ethical leaders display strong moral character having a strong concern for people. Further, they pay immense attention to environmental compliance. Leaders are usually assumed as legitimate role models for normative behaviors [25]. Unlike unethical leaders, ethical leaders are supposed to be more credible in their followers’ eyes and hence are considered as more suitable and attractive role models to follow. As per SLT, employees are more inclined towards ethical leaders for learning desired and appropriate behaviors [22]. In organizational behavior, leaders are vital and indispensable drivers and motivators [48]. The vision of leadership articulates the strategies of the organization, and the behaviors of leaders set examples for followers to behave in any desired and specific direction [49]. Leaders may support, help, and encourage employees to attain their own and the environmental goals of an organization. Ethical leaders are supposed to be effective role models as there is no difference between their said words and actions; hence, they can strongly influence the organization’s ethical climate by developing trust [42]. SLT also emphasizes vicarious learning, along with direct observation [32]. Ethical leadership strongly influences the employees’ ethical behaviors as all moral actions of ethical leaders are visible in the workplace [25,34]. Before acting upon the rules, regulations, and policies, employees need to perceive and interpret their work environment [36]. Ethical leaders put extra efforts into making their organizational climate green by encouraging and motivating employees via the performance of such behaviors being rewarded and cherished. Hence, leaders are supposed to be the explanatory filters of the organizational policies and the employees’ procedures. Based on these policies and practices that are presented to the employee, leaders may eventually influence employees’ perception regarding climate [29]. Employees’ perceptions of the organization’s rules, regulations, and policies about environmental issues result in a green psychological climate. Hence, when employees psychologically feel that the organization is practicing green behaviors, their personal environmental commitment is enhanced. Accordingly, this study proposes the following hypothesis:

**Hypothesis** **2.** *The relationship between ethical leadership and employees’ environmental organizational commitment is mediated by green psychological climate*.

### 2.4. Employees’ Harmonious Environmental Passion, Environmental Commitment and Green Behavior

The harmonious environmental passion of employees lead to EGB, which is a particular type of pro-environmental behavior in the work setting and is explained as “scalable actions and behaviors that employees engage in that are linked with and contribute to or detract from environmental sustainability” [50] (p. 87). EGB can be categorized into rubrics of organizational citizenship behavior, counterproductive work behaviors, and task performance [49,51]. Moreover, Norton [21] posited that activities such as saving energy by switching off the lights when exiting the office, consuming resources effectively, by eliminating unnecessary travel, avoiding waste by editing documents electronically rather than printing them, reporting water leaks in the bathroom, recycling paper, etc., are considered organizational pro-environmental behaviors. Harmonious passion is an energized emotion that inspires employees to make a difference in the workplace and motivates them to perform pro-environmental activities. In the specific context of harmonious environmental passion, employees engage in activities at the workplace that result in the protection of the environment. Moreover, engaging in these activities results in positive emotions, such as happiness and joy, which positively affect employees’ tendency to adopt green behaviors [22]. According Paillé and Mejía-Morelosb [52] report that more satisfied and committed employees in their jobs display a greater number of green behaviors, for example, by conserving and working sustainably. Employee commitment is important as it directs the employees to attain the specific goals of the organization. Thus, in the environment’s particular context, employees who exhibit environmental commitment will execute a more significant number of green behaviors. Therefore, the present research proposes the following hypothesis:

**Hypothesis** **3.** *There is a positive relationship between employees’ harmonious environmental passion and employee green behavior (3a) and between employees’ environmental commitment and employee green behavior (3b)*.

### 2.5. Serial Mediation of a Green Psychological Climate and Employees’ Harmonious Environmental Passion on the Indirect Effect of Ethical Leadership on Employee Green Behavior

Leadership is an important predictor of EGB as leaders reveal their values through their behaviors [52]. In the sections above, the positive association between ethical leadership and EGB has been extensively discussed. Moreover, these sections revealed that green psychological climate and employees’ harmonious environmental passions play significant roles in explaining the relationship between the two constructs. Specifically, green psychological climate is considered an important contextual factor that influences EGB [53]. As mentioned, green psychological climate plays a significant mediating role in the ethical leadership-employees’ relationship to harmonious environmental passion. Therefore, a leader with a strong pro-environmental attitude will put forth extra effort to make the climate of the organization greener. When employees receive signals regarding the climate of the organization, particularly that practicing green behaviors is strongly encouraged, employees become more passionate about displaying green behaviors. Accordingly, the following hypothesis is proposed:

**Hypothesis** **4.** *Green psychological climate and employees’ harmonious environmental passion sequentially mediate the relationship between ethical leadership and employee green behavior*.

### 2.6. Serial Mediation of a Green Psychological Climate and Employees’ Environmental Commitment on the Indirect Effect of Ethical Leadership on Employee Green Behavior

Ethical leadership promotes a green psychological climate, and a green psychological climate mediates the relationship between ethical leadership and employees’ environmental commitment. The psychological climate is referred as the shared perceptions of employees regarding organizational policies and practices [35,36]. Environmental commitment is a frame of mind that denotes responsibility to and a sense of attachment to environmental issues in the workplace [47]. Moreover, employees with environmental commitment exhibit a greater number of green behaviors. Thus, it can be expected that ethical leaders will make their workplace climate greener. Furthermore, a green psychological climate enhances employees’ environmental commitment, as they strive to cope with the organization’s environmental policies, both mentally and physically, by displaying green behaviors. Thus, the following hypothesis is proposed:

**Hypothesis** **5.** *Green psychological climate and employees’ environmental commitment sequentially mediate the relationship between ethical leadership and employee green behavior*.

### 2.7. Moderation of Leaders’ Pro-Environmental Attitudes

In previous research, we discussed that people inclined towards environmental safety are presumed to have pro-environmental attitudes and concerns regarding the natural environment [54]. Moreover, there is a positive association between pro-environmental attitude and pro-environmental behavior [55,56]. Cordano and Frieze [57] pointed out that managers will display more suitable actions and behaviors with a pollution prevention attitude and be more engaged in pollution-preventing behaviors. Furthermore, it was found that managers with a pro-environmental attitude strongly influence the implementation of management protection programs [58]. Bissing-Olson et al. [59] noted that people with pro-environmental attitudes are worried about the environment and are more likely to perform activities that can improve the environment. Thus, leaders’ pro-environmental attitude can immensely influence a manager’s intention to implement environmental management programs in the workplace [59]. Leaders’ uniformity between stated values and displayed values is also critically important for increasing employees’ commitment to adopt green behaviors [60]. Organizational leaders are considered role models due to their well-defined status, power, and position. Therefore, if they show consistency and commitment in performing green behaviors, displaying environmental leadership, and communicating green policies, they signal to their employees that these behaviors are appreciated and anticipated in the organization. Consequently, followers learn that performing green behaviors will lead to the behaviors desired in their workplace, and their motivation to engage in these behaviors increases tremendously [22,61,62]. Thus, when organizational leaders are ethical and display high pro-environmental attitudes, employees’ perceptions of organizational attributes and behavioral norms are increased and lead to employees’ harmonious environmental passion and employees’ environmental commitment, ultimately translating to EGB. Accordingly, the following hypothesis is proposed:

**Hypothesis** **6.** *Group level leaders’ pro-environmental attitudes moderate the indirect effect of ethical leadership on EGB through the serial mediation of green psychological climate and employees’ harmonious environmental passion (H6a) and green psychological climate and employees’ environmental commitment (H6b), and the effect will be stronger when the leaders’ pro-environmental attitudes are high compared to when they are low*.

## 3. Materials and Methods

The present research takes a post-positivist perspective, and thus, a quantitative research design is employed with a deductive approach to theory development. Specifically, this study establishes the relationship between ethical leadership and EGB and concludes that the relationship is a measurable phenomenon. It also investigates the factors that can explain this relationship by developing theoretical models based on cause and effect as underpinned by social learning theory. This multi-level, multisource, mono-method quantitative study employs a time-lagged design to collect data by following a survey strategy. Further, multiple units of analysis are considered as this study is based on multisource and multi-level research. For instance, ethical leadership and leaders’ pro-environmental attitude are group-level variables, and therefore the unit of analysis is a group. Variables such as green psychological climate, employees’ environmental commitment, employees’ harmonious environmental passion, and EGB are individual-level variables and are therefore analysed as individual units.

### 3.1. Data Collection and Sampling Strategy

We followed a two-stage sampling process. In Punjab, around 70 universities are recognized by the Higher Education Commission of Pakistan and 22 hospitals recognized by the Pakistan Medical and Dental Council. We randomly selected six universities (3 public and 3 private) and four hospitals (2 public and 2 private) in the first stage. We requested the participation of the top management in the surveys by explicitly communicating our research objectives. Finally, we obtained approval from four organizations, two universities (one public and one private), and two hospitals (one public and one private) located in the two large metropolitan cities.

We signed ethics and confidentiality agreements with these organizations and also promised to keep anonymity. Then, confidentiality and ethics assurance forms were signed, and privacy guarantees were extended. With the assistance of the participating organizations’ human resource departments, we identified 92 workgroups with a mean size of 10.5 employees (n = 958), and each workgroup had an exclusive manager/leader/supervisor. In 64 work units with more than five people, five employees were randomly selected. For the remaining 28 work units with five employees, each individual was targeted because no groups had fewer than five employees. The planned sample was 460 employees reporting to 92 leaders. The present research objectives were communicated to all the selected individuals, who were then requested to take part in the study by completing the survey. Participants were ensured of confidentiality and anonymity of the information linked to the surveys. To avoid common method biases for the predictor and criterion variables associated with one-time data collection, we employed a time-lagged design and collected data in three different periods by administering four surveys (three from employees and one from leaders) three weeks apart between December 2019 and January 2020.

The first employee survey (at time 1) assessed the employees’ perceptions of their leaders’ ethical values and employees’ harmonious environmental passion. The respondents reported on their immediate leader and provided a six-digit code to match the following survey data. This survey distributed self-administrated questionnaires to the planned sample (i.e., 460 employees), and 425 questionnaires (92.4%) were completed and returned. The second employee survey (at time 2) assessed the green psychological climate and EGB. In this round, the leader survey measured the leaders’ pro-environmental attitude. This time 425 self-administrative questionnaires were distributed among those who participated in the first survey, and 410 (96.5%) completed questionnaires were returned. Side by side, in the leader survey, 92 questionnaires were distributed among the employees’ immediate leaders, and 84 leaders (91.3%) completed the questionnaires. Finally, the third employee survey (at time 3) captured employees’ environmental commitment. We distributed 410 self-administrated questionnaires among the respondents who had completed the second employee survey, and 400 (97.6%) completed questionnaires were returned. After a preliminary data screening and cleaning, 80 leaders’ questionnaires and 400 employees’ questionnaires were deemed completed in all aspects and appropriate for further analysis. Overall, the sample size represents about 42% of the sampling frame; the response ratio per item is 6.6, well above five. Above 300 cases are considered suitable for factor analysis [63]. We have six (a small number) well-determined factors/constructs for which responses per construct are very high (i.e., 66.7). Thus, the sample size is quite reasonable in this study.

The overall response rates were 87% for the leader survey and 86% for the employee survey. The sample characteristics (target organization, sector, and gender) for the 60 non-respondent/incomplete surveys were almost the same as the 400 final useable surveys. In other words, the overall non-response was similar to those of the four organizations, two sectors, and the gender grouping. Likewise, non-response bias comparing early and late respondents was not significant. The survey procedures detailed above enabled us to achieve this high response rate, which is similar to that of other studies conducted in Pakistan [64,65]. Overall, we can safely claim that non-response bias did not pose any threat to our results’ representativeness.

Of the actual sample of 400, about 61% were public sector employees; about 69% were males; a well-educated sample, about 97%, reported a formal education of 18 years or above. The employee representation for the four age categories of 20–25, 26–30, 31–40, and above 40 years was 1.5%, 12.5%, 51.5%, and 34.5%, respectively. In terms of employees’ experience, four experience categories of 1–3, 4–7, 8–12, and above 12 years, the respective percentage representation was 17%, 23.8%, 32.8%, and 26.5%. The employee sample was well-experienced; about 67% had more than seven years of work experience. On the other hand, of the actual sample of 80 leaders, about 86% were male; about 71% reported a formal education of 18 years or above. The leader representation in the same four age brackets was 32.5%, 3.7%, 26.3%, 37.5%, respectively. The respective percentage representation of leaders in the same four experience categories was 2.5%, 38.7%, 43.7%, and 15%. Thus, the leader sample was also well-educated; about 59% had more than seven years of work experience.

### 3.2. Measures

This study relies on well-established measures widely used in the literature to estimates the six constructs. Ethical leadership was measured using Brown et al.’s [30] ten items scale (i.e., Makes fair and balanced decisions). The respondents responded to items on a five point Likert scale (1 = never to 5 = every time) with 0.96 Cronbach’s alpha value. This scale is also validated by the existing research; for instance Ofori [66], and Pasricha and Rao [67] found a Cronbach’s alpha value of 0.92 and 0.94, respectively. Leaders’ pro-environmental attitude was measured on a 15-item five point Likert type scale (1 = strongly disagree, 5 = strongly agree) [68]. The reliability of this scale was indicated by a Cronbach’s alpha of 0.70. The following is a sample item from this instrument: “Humans are seriously abusing the environment.” A similar scale was used by Tian et al. [69] and noticed a Cronbach’s alpha value of 0.83. The five-item scale developed by Norton et al. [70] was utilized to measure green psychological climate on a Likert scale ranging from 1 (strongly agree) to 5 (strongly disagree). Cronbach’s alpha was 0.93. The following is an example item from this instrument: “My company is worried about its environmental effects.” Khan et al. [71] employed a similar scale, with a Cronbach’s alpha value of 0.89. Employees’ harmonious environmental passion was measured by using the 10-item scale [22]. Cronbach’s alpha for this scale was 0.90. Each item was rated on a 5 point scale (1 = strongly disagree; 5 = strongly agree). The following is a sample item from this instrument: “I am passionate about the environment.” In the same vein, Afsar [72] found the Cronbach’s alpha for this scale was 0.87.

The eight-item scale of Raineri and Paillé [47] was used to evaluate employees’ environmental commitment, and each item was measured on a 6-point scale ranging from 1 (strongly disagree) to 6 (strongly agree). Cronbach’s alpha was 0.83. The following is a sample item from this instrument: “I feel personally attached to the environmental concerns of my company.” Cronbach’s alpha value of 0.91 was reported for this scale by [73]. EGB was measured using the 13-item scale developed by Graves et al. [60]. Each item was assessed on a five-point scale ranging from 0 (not at all) to 4 (frequently if not always). Cronbach’s α value was 0.83. The following is a sample item from this instrument: “I recycle and reuse materials”. Norton et al. [70] employed a similar scale with a Cronbach’s alpha value of 0.92.

This research’s control variables consist of employees’ and immediate leaders’ education, gender, tenure, and age, because of their possible influence on leaders’ attitudes and subordinates’ perception and behaviors. To control for the possibility of organizations’ unique effect, we also incorporated an organization dummy variable, because the data were collected from four organizations, and every dataset can have distinctive features. Further, the effect of employees’ environmental commitment was also controlled to keep the underlying model simple. Controlling for these variables would have minimized the possible effect of other variables not included (omitted variables) in the study when explaining EGB.

### 3.3. Analysis Strategy

**Figure 1:**
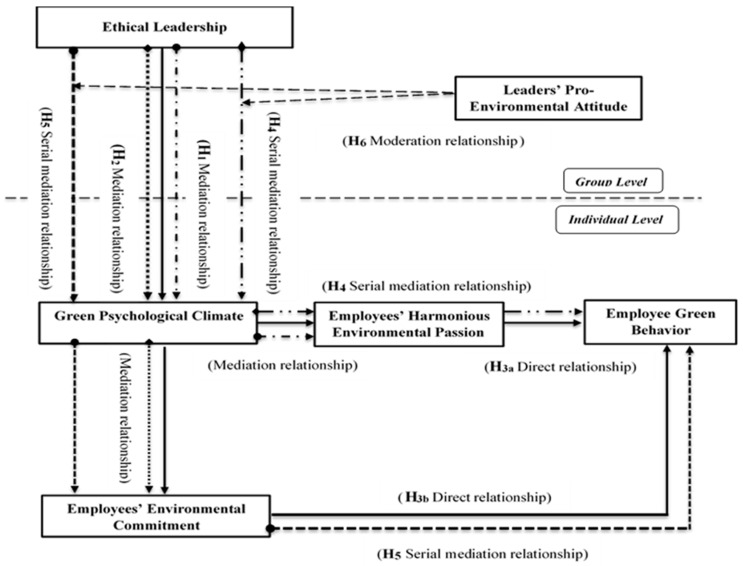
Hypothesized Model.

The present study employed a multi-level theoretical framework and utilized nested data to examine the underlying model. As presented in Figure 1, this research proposed that leaders’ pro-environmental attitude and supervisory ethical leadership are at the group-level. In contrast, subordinates’ green psychological climate, harmonious environmental passion, environmental commitment, and green behavior are at the individual level. Leaders’ pro-environmental attitude was estimated at the group-level by directly taking data from the leaders specified in the leader survey (time 2). To create group-level ethical leadership, this research aggregated the employees’ individual-level responses to rate their immediate leader, as per Chan’s [74] typology of the direct consensus model. Such aggregation has been established and supported in the existing literature [75]. Moreover, to validate and support the aggregation of individual-level responses of ethical leadership at a group level, this study estimated the intraclass correlation (ICC) and Rwg (j) values by following the existing literature [76]. The values were found to be within the acceptable range (ICC1 = 0.30, ICC2 = 0.42 and Rwg (j) = 0.70), validating the aggregation accompanied in this research and were consistent with the existing literature that has also reported on this type of aggregation [77,78,79,80]. Further, the validity of the constructs and model fit indices were assessed by performing multi-level confirmatory factor analysis in MPlus. MPlus provides novel approaches to data analysis that includes longitudinal, multi-level, and cross-sectional data. As this research sought to examine multi-level data, MPlus was employed. The model fitness was assessed by employing frequently used indicators, including the Tucker-Lewis index (TLI), comparative fit index (CFI), Chi-square/degree of freedom, root mean square error approximation (RMSEA), and standardized root mean square residual (SRMR). It was found that CFI = 0.970, TLI = 0.954, chi-square/degree of freedom = 1.811, RMSEA = 0.054, SRMRwithin = 0.039, SRMRbetween = 0.021. The constructs’ validity and reliability were confirmed by Cronbach’s alpha, composite reliability, average variance extracted, and maximum shared variance. Further, descriptive and inferential analyses were conducted. In addition, multi-level path analysis was performed to test the hypotheses by employing multi-level modeling in MPlus.

This study employed various preventive measures to evade common method biases. The respondents were not asked to show their titles, names, departments, and their organization name to avoid social desirability bias. Further, it is also noticed that less social responsibility can be achieved by enhancing respondents’ anonymity. The research also promised and maintained the privacy and confidentiality of the respondents’ responses in every facet. It was exhaustively explained to the respondents’ that their responses will only be utilized for academic research and will not be disclosed to anyone. Thus, this leads us to overcome self-report biases. Moreover, the researcher requested the respondents to honestly respond to the questions, as honest responses minimize biased responses. This study employed time-lag design to collect data from multiple sources, reducing common method biases. For instance, the data for predicator (ethical leadership) and criterion (EGB) variables were collected in two waves by conducting two different surveys. These above-mentioned practical remedies are noted to reduce the problem of common method biases. Yet, this research also used familiar statistical tools to examine the data for common method biases, including Harman’s one-factor test [81], and the common latent factor procedure of Podsakoff et al. [82]. The existing research preferred common latent factors as a well-recognized and preferred tool over Harman’s one factor, because it has various advantages, including simple operating and assessment procedures. We used both tools and found that single factor extraction was 22% by employing Harman’s one-factor test, and 16% variance was produced in common latent factor analysis. These values were found to be in the acceptable range [82,83], and thus it was noted that common method biases were not the issue in this research.

## 4. Data Analysis and Results

The reliability and validity of all constructs are reported in Table 1. Cronbach’s alpha values were satisfactory and exceeded the threshold value of 0.70 (0.96 for ethical leadership to 0.70 for leaders’ pro-environmental attitudes). Thus, internal consistency was confirmed for all variables [70]. Likewise, the results were in the acceptable range for average variance extracted (AVE), composite reliability (CR), and maximum shared variance (MSV) as the values were above 0.70 for CR and above 0.50 for AVE, and the MSV was less than the AVE for all constructs. Descriptive statistics and bivariate correlation results are reported in Table 2. The central tendency and data dispersion are reported as the means and standard deviation. Moreover, normality is tested using Skewness and Kurtosis.

The mean values were as follows: ethical leadership = 4.20, green psychological climate = 4.22, EGB = 3.09, employees’ harmonious environmental passion = 4.01, employees’ environmental commitment = 4.37, and leaders’ pro-environmental attitude = 4.33. Furthermore, all variables’ standard deviation values were in the normal range (not too high and not too low) and revealed little dispersion in the dataset. With respect to the normality of variables, the Skewness and Kurtosis values were within the acceptable range (from +2.58 to −2.58) [84]. We also found a bivariate correlation among study variables in the supposed direction. Thus, the data were appropriate for further analysis.

Table 3 reports the direct effects of the study variables. The regression coefficient for group-level ethical leadership and individual-level green psychological climate was 0.173, *p* < 0.05; for the group-level ethical leadership and individual-level employees’ harmonious environmental passion, it was 0.155, *p* < 0.05; and for the group-level ethical leadership and individual-level EGB, it was 0.155, *p* < 0.05. These results indicate the positive and statistically significant effect of ethical leadership on green psychological climate, employees’ harmonious environmental passion, and EGB. Thus, by maintaining all other variables constant, if ethical leadership increases by one unit, then green psychological climate, employees’ harmonious environmental passion, and EGB increase by 0.173, 0.155, and 0.151 units, respectively. However, we found a statistically insignificant positive impact of ethical leadership on employees’ environmental commitment, 0.062, *p* > 0.05. Further, there were positive and statistically significant effects of individual-level green psychological climate on individual-level employees’ harmonious environmental passion, 0.504, *p* < 0.01, and of individual-level green psychological climate on individual-level employees’ environmental commitment, 0.308, *p* < 0.01. The positive and statistically significant effects of employees’ harmonious environmental passion on EGB, 1.070, *p* < 0.01, and employees’ environmental commitment on EGB, 0.866, *p* < 0.01 were also found. Hypothesis 3 proposed a positive relationship between employees’ harmonious environmental passion and EGB (3a) and between employees’ environmental commitment and EGB (3b). Thus, Hypothesis 3 is supported.

Further, Table 4 presents the cross-level mediation and serial mediation effects. Hypothesis 1 posited that the relationship between group-level ethical leadership and individual-level employees’ harmonious environmental passion is mediated by individual-level green psychological climate. Accordingly, we found a statistically significant positive mediation effect (EL → GPC → EHEP) 0.087 (95% CI (0.021, 0.154)). The direct effect of group-level ethical leadership on employees’ harmonious environmental passion is reported in Table 3 to be 0.155, *p* < 0.05. The total effect of ethical leadership on employees’ harmonious environmental passion was estimated to be 0.242 (EL → EHEP + EL → GPC → EHEP = 0.155 + 0.087 = 0.242). Thus, the mediation effect of green psychological climate was 35.9% (0.087/0.242 = 0.359). Hence, Hypothesis 1 is accepted.

Hypothesis 2 posited that the relationship of group-level ethical leadership and individual-level employees’ environmental commitment is mediated by individual-level green psychological climate. Accordingly, we found a statistically significant positive mediation effect (EL → GPC → EEC) 0.053 (95% CI [0.011, 0.096]). The direct effect of group-level ethical leadership on employees’ environmental commitment, reported in Table 3, was statistically insignificant at 0.062, *p* > 0.05. Thus, the full mediation of green psychological climate is shown, and Hypothesis 2 is accepted.

Hypothesis 4 specified that green psychological climate and employees’ harmonious environmental passion sequentially mediate the relationship between ethical leadership and EGB. Accordingly, we found a statistically significant positive cross-level mediation effect (EL → GPC → EHEP →EGB) 0.093 (95% CI [0.016, 0.170]). The direct effect of group-level ethical leadership on EGB, reported in Table 3, was 0.151, *p* < 0.05. The total effect of ethical leadership on EGB was estimated to be 0.244 (EL → EGB + EL → GPC → EHEP → EGB = 0.151 + 0.093 = 0.244), and thus, the serial mediation effect was 38.1% (0.093/0.244 = 0.381). Accordingly, Hypothesis 4 is accepted. Furthermore, with respect to Hypothesis 5, we found that green psychological climate and employees’ environmental commitment sequentially mediated the relationship between ethical leadership and EGB. Accordingly, we found a statistically significant positive cross-level mediation effect (EL → GPC → EEC → EGB) 0.045 (95% CI [0.009, 0.081]). The direct effect of group-level ethical leadership on EGB, reported in Table 3, was 0.151, *p* < 0.05. The total effect of ethical leadership on EGB was estimated to be 0.196 (EL → EGB + EL → GPC → EEC → EGB = 0.151 + 0.045 = 0.196). Therefore, the portion of serial mediation effect was 22.9% (0.045/0.196 = 0.229). Accordingly, Hypothesis 5 is supported.

Further, to test the boundary condition of leaders’ pro-environmental attitude on the indirect effects of group-level ethical leadership and EGB, we first investigated the interaction of group-level ethical leadership and leaders’ pro-environmental attitude (see Table 5). We found a positive and statistically significant effect of the interaction term on green psychological climate (0.291, *p* < 0.01). Besides, Table 5 reported the moderated mediation results with a bootstrap at a 95% CI.

**Figure 2:**
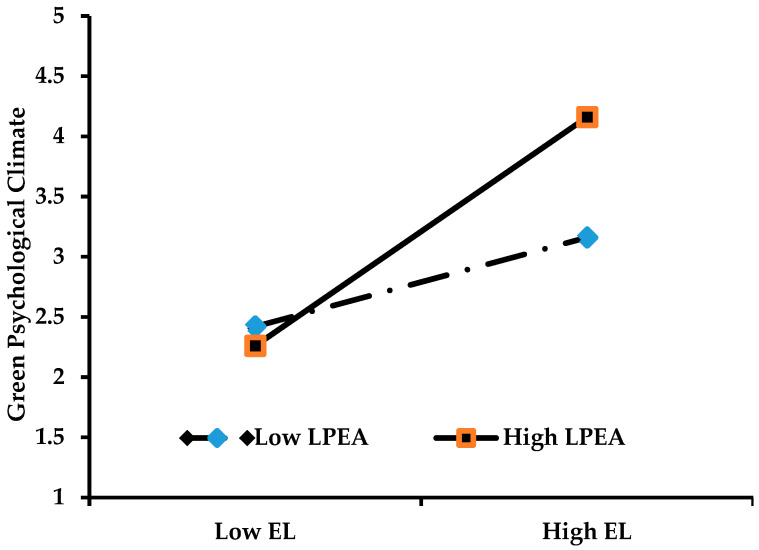
Moderation of Leaders’ Pro-environmental Attitude.

The results show that the values for the serial mediation of green psychological climate and employees’ harmonious environmental passion with the moderation of leaders’ pro-environmental attitude were 0.157 (95% CI (0.072, 0.242)), and 0.093 (95% CI (0.016, 0.170)) without the moderation of leaders’ pro-environmental attitude. Thus, it is evident that the relationship is strengthened by the contingency of leaders’ pro-environmental attitude. Similarly, the values for the serial mediation of green psychological climate and employees’ environmental commitment with the moderation of leaders’ pro-environmental attitude were 0.077 (95% CI (0.004, 0.150)), and 0.045 (95% CI (0.009, 0.081)) without the moderation of leaders’ pro-environmental attitude, as reported in Table 4. Accordingly, this relationship is also strengthened by the contingency of leaders’ pro-environmental attitude. Hypothesis 6 stated that group-level leaders’ pro-environmental attitude moderates the indirect ethical leadership–EGB relationship through the serial mediation of green psychological climate and employees’ harmonious environmental passion (H6a) and green psychological climate and employees’ environmental commitment (H6b), and that the impact is stronger when the leaders’ pro-environmental attitude is high compared to when it is low. Therefore, hypotheses 6a and 6b are accepted. Moreover, for the better understanding of leaders’ pro-environmental attitude contingency effect on the indirect effect of ethical leadership on EGB, we plotted the moderation effects on the ethical leadership and green psychological climate relationship, as shown in Figure 2, which translates into employees’ harmonious environmental passion and employees’ environmental commitment, and in-turn leads to EGB. Figure 2 shows that the relationship between ethical leadership and green psychological climate is stronger when leaders’ pro-environmental attitude is high compared to when it is low.

## 5. Discussion

According to Lu et al. [85], promoting and inculcating employee green behavior is the central pathway for organizations to promote sustainable development and to recognize their social responsibility. The present study intended to examine a multi-level mechanism that relates group-level ethical leadership to individual-level EGB with the serial and multi-mediation of green psychological climate and employees’ harmonious environmental passion, and through green psychological climate and employees’ environmental commitment. Moreover, the importance of this study was further improved with the contingency of leaders’ pro-environmental attitude. The literature suggests that leadership is an indispensable determining factor of organizational climate [85], as leaders perform an essential role in shaping this climate [34].

Consistent with this premise, previous research has noticed the positive impact of ethical leadership on employees’ ethical and pro-social behaviors. The literature has further concluded that ethical leadership minimizes employees’ unethical behaviors [71,86,87], and is significantly related to various employee work outcomes [88]. Ethical leaders establish the moral tendency of organizations [44]. Therefore, employees’ workplace attitudes and behaviors are influenced by ethical leadership [89]. Further, organizational behavior research has validated the affirmative consequences of ethical leadership; but there exists limited research on the effects of ethical leadership on green psychological climate. Early attempts on this relationship include Khan et al. [71] and Saleem et al. [79], who found a positive effect of ethical leadership on green psychological climate. Using employees from a sample of hospitals and universities, this study noted the positive impact of ethical leadership on green psychological climate. Following the social learning theory of Bandura [32], we proposed that ethical role modelling comes from leaders who influence the climate by displaying behaviors and establishing trust that align with their words. Moreover, this study advocated that ethical leaders perform an imperative role in modifying and shaping the organization’s climate. Therefore, this research’s findings not only align with the existing studies, revealing the positive effect of ethical leadership on green psychological climate, but also progress the debate on the relationship between ethical leadership and green psychological climate at multiple levels.

Second, this research found that the mediation of green psychological climate indirectly affects group-level ethical leadership on employees’ harmonious environmental passion. The existing literature indicates that leaders can evoke emotions in followers and that employees’ harmonious environmental passion is a positive emotion that encourages employees to engage in pro-environmental behaviors [22], as employees are more likely to perform and be passionate about behaviors that are vital to the organizations as well as of social importance. Further, employees will automatically harmonize according to the actions and expressions of their leaders. This phenomenon, known as emotional contagion, occurs between leaders and followers and is considered an important part of the leadership process [40]. Hence, this emotional contagion ignites employees’ harmonious environmental passion [22]. Although limited research has been conducted on employees’ harmonious environmental passion, our results are according with the existing literature that documented positive workplace outcomes as a result of ethical leadership [90], as well as a positive association between green psychological climate and employees’ harmonious environmental passion [91,92]. Third, we tested and noted a positive and statistically significant indirect effect of green psychological climate mediation of group-level ethical leadership on employees’ environmental commitment. Although numerous studies have examined employee commitment’s determinants and outcomes, according to Paillé and Valéau [73] employees’ environmental commitment has recently resurfaced in work environment research. Thus, our results are not only in accordance with the existing research on employees’ environmental commitment [93], but they extend the field by uncovering the impacts of ethical leadership and green psychological climate on employees’ environmental commitment, an area that has not been jointly considered to date. Therefore, we call for more research to verify the results of the present study.

Fourth, this research identified a positive link between employees’ harmonious environmental passion, employees’ environmental commitment, and EGB, a finding that is consistent with the existing research [22,71,93]. Finally, this study examined the boundary conditions of leaders’ pro-environmental attitude on the indirect relationship between ethical leadership and EGB. We further noted that the relationship between ethical leadership and green psychological climate, along with the moderating effect of leaders’ pro-environmental attitude that translates into employees’ harmonious environmental passion and employees’ environmental commitment, ultimately indicates that the relationship with EGB has not been jointly investigated thus far.

However, previous studies have discussed that employees eventually follow the directions of their leaders and top management and will act in accordance with management’s desires [94]. As the leader’s vision may ultimately lead to the strategies adopted by the organization, the leader’s attitude towards the protection of the environment may ultimately encourage employees to work toward environmentally sustainable goals [50]. Moreover, earlier research has documented that the extent to which the leader participates in and talks about protecting the environment affects the degree to which followers will practice green and eco-friendly behaviors [35]. In support of this premise, we concluded that leaders’ pro-environmental attitude strengthened the indirect effect of the relationship between ethical leadership and EGB. Hence, we call for further research to validate our results.

### 5.1. Research Implications

Theoretically, this research contributed to seven knowledge domains: ethical leadership [31,85]; green psychological climate [37,95]; employees’ harmonious environmental passion [22,40]; employees’ environmental commitment [45,46,47]; EGB [19,50,68,71]; social learning theory [87,96]; and leaders’ pro-environmental attitude [52], and further extended the literature on the consequences of ethical leadership and precursors of EGB at multiple levels. For instance, ethical leaders can establish rules, regulations, and practices that make the climate of the organization greener. Furthermore, our model enriches the literature with the contribution of leaders’ pro-environmental attitude as a contingency factor. Our study finds that a pro-environmental attitude strengthened the ethical leadership–EGB relationship. Finally, we have extended the previous research by arguing that leaders’ behavioral signals regarding environmental protection further support employee engagement in adopting green behaviors [90,97].

Practically, this study offers various insights for managers and organizations. For instance, the present research’s fundamental idea is closely assimilated with the status quo and the organizations’ eco-friendly sustainable development plans. This research concentrates on organizational leaders’ and employees’ roles in implementing eco-friendly practices and protecting natural resources through energy saving, reducing waste, and recycling. The present study offers a good road map for organizational practitioners to develop their employees into eco-friendly activists to benefit sustainable growth. In this regard, EGB is considered significant in the accomplishment of an organization’s eco-friendly practices. Organizations can attain EGB by monitoring ethical leadership and leaders’ pro-environmental attitudes. Leaders and top management should nourish the green psychological climate if they want their followers to perform green behaviors. It is a usual practice that employees follow the same directions provided by the management and their leaders and opt for the same behaviors that they observe in their immediate leaders. Employees should be given clear signals, and this sort of vision should be enunciated by the leaders and organization to stimulate the significance of sustainable environmental goals at the workplace. It can be said precisely that to attain sustainable organizational goals, organizations should focus on managers’ ethical behaviors. Managers should inculcate green behaviors by setting GPC via role modeling. Ethical leaders and pro-environmental attitudes may play a significant role in promoting GPC in the workplace. Human resource management must pay attention to the recruitment processes by integrating sustainable strategies in overall operations. During interviews, environment and ethics-related questions could be observed, and applicants can be asked to evaluate their ethical values and environmental commitment. Questions can be asked to observe their environmental harmonious passion and how passionate they are in practicing environmentally friendly practices. Finally, EGB can be promoted in organizations by providing opportunities to take part in organizational sustainability policies.

### 5.2. Limitations and Future Directions

Despite the outstanding findings pointed out in this research, some limitations should be considered that simultaneously offer insights for future areas of research. For instance, we only considered the boundary condition of leaders’ pro-environmental attitude that strengthens the indirect effect of ethical leadership on EGB. Future research should consider other contextual or individual differences that can affect the indirect relationship between ethical leadership and EGB, such as perceived organizational support and employee environmental awareness. Further, we formulated and examined the mechanisms to derive EGB. It will be interesting to discover the outcomes of EGB, such as green innovations. Moreover, given that the sample organizations for this research are from the services sector, future research should also focus on other industries and jobs. Although we included five control variables to test our hypotheses, we cannot rule out omitted variable bias in our estimates. Future studies may consider conscientiousness, perceived organizational support, and pro-social motivation in this category.

## 6. Conclusions

In the growing EGB research, we attempted to formulate and investigate an influencing mechanism of ethical leadership towards EGB along with the contingency of leaders’ pro-environmental attitude. A multi-level moderated mechanism encompassing social learning theory has been employed to develop a multilevel model for this research, including ethical leadership, green psychological climate, employees’ environmental commitment, employees’ harmonious environmental passion, and contingency effect of leaders’ pro-environmental attitude as the antecedents of EGB. It was founded that ethical leadership is a significant predictor of a green psychological climate that ultimately turns into employees’ environmental commitment, and employees’ harmonious environmental passion which in turn translate into EGB. Our study has revealed that ethical leadership makes such a climate in the organization that promotes green behaviors at the workplace. The success of organizations’ environmental strategies depends upon the free and spontaneous pro-environmental behaviors of the employees. We have strongly emphasized that ethical leaders’ having a pro-environmental attitude encourages employees to perform green behaviors in the workplace.

## Figures and Tables

**Table 1:** Reliability and validity of scales.

Variable	Items	Alpha	CR	AVE	MSV
Ethical Leadership	10	0.96	0.81	0.57	0.32
Green Psychological Climate	5	0.93	0.91	0.50	0.29
Employee Green Behavior	13	0.83	0.72	0.59	0.43
Employees’ Harmonious Environmental Passion	10	0.90	0.70	0.60	0.51
Employees’ Environmental Commitment	8	0.83	0.91	0.63	0.41
Leaders’ Pro-environmental Attitude	15	0.70	0.83	0.54	0.37

Notes: Alpha = Cronbach Alpha, CR = composite reliability, AVE = average variance extracted, MSV = maximum shared variance.

**Table 2:** Correlation matrix of the study variables.

Variable	Mean	Range	SD	Ske	Kur	1	2	3	4	5
1. EL	4.20	1–5	0.57	−2.05	1.98	1				
2. GPC	4.22	1–5	0.75	−1.60	2.08	0.22 **	1			
3. EGB	3.09	0–4	0.52	−1.31	1.38	0.35 **	0.39 **	1		
4. EHEP	4.01	1–5	0.56	2.00	2.02	0.16 **	0.55 **	0.63 **	1	
5. EEC	4.37	1–6	0.51	−0.97	0.53	0.40 **	0.41 **	0.57 **	0.37 **	1
6. LPEA	4.33	1–5	0.24	0.37	0.70	0.03	0.05	0.02	0.04	0.01

Notes: ** *p* < 0.01, Ske = Skewness, Kur = Kurtosis, SD = Standard deviation, EL = Ethical leadership, GPC = Green psychological climate, EGB = Employee green behavior, EHEP = Employees’ harmonious environmental passion, EEC = Employee environmental commitment, LPEA = Leaders’ pro-environmental attitude.

**Table 3:** Summary of direct effects.

	Estimates	95% CI	Remarks
Group → Individual			
EL → GPC	0.173 *	(0.040, 0.307)	
EL → EHEP	0.155 *	(0.060, 0.250)	
EL → EEC	0.062	(−0.035, 0.160)	
EL → EGB	0.151 *	(0.087, 0.215)	
Individual → Individual			
GPC → EHEP	0.504 **	(0.453, 0.555)	
GPC → EEC	0.308 **	(0.234, 0.382)	
EHEP → EGB	1.070 **	(0.457, 1.683)	Supported (H_3a_)
EEC → EGB	0.866 **	(0.495, 1.237)	Supported (H_3b_)

Notes: CI = confidence interval, ** *p* < 0.01, * *p* < 0.05, EL = Ethical leadership, GPC = Green psychological climate, EGB = Employee green behavior, EHEP = Employees’ harmonious environmental passion, EEC = Employee environmental commitment, LPEA = Leaders’ pro-environmental attitude.

**Table 4:** Summary of mediation effects.

	Estimates	95% CI	Remarks
Group → Individual → Individual			
EL → GPC → EHEP	0.087 *	(0.021, 0.154)	Supported (H_1_)
EL → GPC → EEC	0.053 *	(0.011, 0.096)	Supported (H_2_)
Group → Individual → Individual → Individual			
EL → GPC → EHEP → EGB	0.093 *	(0.016, 0.170)	Supported (H_4_)
EL → GPC → EEC → EGB	0.045 *	(0.009, 0.081)	Supported (H_5_)

Notes: CI = confidence interval. * *p* < 0.05. EL = Ethical leadership, GPC = Green psychological climate, EGB = Employee green behavior, EHEP = Employees’ harmonious environmental passion, EEC = Employee environmental commitment, LPEA = Leaders’ pro-environmental attitude.

**Table 5:** Summary of moderation effects.

	Estimates	95% CI	Remarks
Group * Group → Individual			
EL * LPEA → GPC	0.291 **	(0.061, 0.522)	
Group * Group → Individual → Individual → Individual			
EL * LPEA → GPC → EHEP → EGB	0.157 **	(0.072, 0.242)	Supported (H_6a_)
EL * LPEA → GPC → EEC → EGB	0.077 *	(0.004, 0.150)	Supported (H_6b_)

Notes: CI = confidence interval, ** *p* < 0.01, * *p* < 0.05. EL = Ethical leadership, GPC = Green psychological climate, EGB = Employee green behavior, EHEP = Employees’ harmonious environmental passion, EEC = Employee environmental commitment, LPEA = Leaders’ pro-environmental attitude.

## Data Availability

The dataset used in this research are available upon request from the corresponding author. The data are not publicly available due to restrictions i.e., privacy or ethical.
